# The Relationship between Leukocyte Mitochondrial DNA Copy Number and Telomere Length in Community-Dwelling Elderly Women

**DOI:** 10.1371/journal.pone.0067227

**Published:** 2013-06-13

**Authors:** Jung-Ha Kim, Hye Kyung Kim, Jae-Hong Ko, Hyoweon Bang, Duk-Chul Lee

**Affiliations:** 1 Department of Family Medicine, Chung-Ang University Healthcare System, Seoul, Korea; 2 Health Promotion Center, Gangnam Severance Hospital, Yonsei University College of Medicine, Seoul, Korea; 3 Department of Physiology, College of Medicine, Chung-Ang University, Seoul, Korea; 4 Department of Family Medicine, Severance Hospital, Yonsei University College of Medicine, Seoul, Korea; University of Valencia, Spain

## Abstract

**Purpose:**

Both telomere length and mitochondrial function are accepted as reflective indices of aging. Recent studies have shown that telomere dysfunction may influence impaired mitochondrial biogenesis and function. However, there has been no study regarding the possible association between telomere and mitochondrial function in humans. Therefore, the purpose of the study was to identify any relationships between mitochondrial and telomere function.

**Methods:**

The present study included 129 community-dwelling, elderly women. The leukocyte mitochondrial DNA copy number and telomere length were measured using a quantitative real-time polymerase chain reaction method. Anthropometric measurement, biochemical blood testing, a depression screening questionnaire using a 15-question geriatric depression scale (GDS-15), and a cognitive function test using the Korean version of the mini mental state examination (K-MMSE) were performed.

**Results:**

Leukocyte mtDNA copy number was positively associated with telomere length (*r*=0.39, *p*=<0.0001) and K-MMSE score (*r*=0.06, *p*=0.02). Additionally, leukocyte mtDNA copy number was negatively correlated with GDS-15 score (*r*=-0.17, *p*=0.04). Age (*r*=-0.15, *p*=0.09), waist circumference (r=-0.16, *p*=0.07), and serum ferritin level (*r*=-0.13, *p*=0.07) tended to be inversely correlated with leukocyte mtDNA copy number. With a stepwise multiple regression analysis, telomere length was found to be an independent factor associated with leukocyte mtDNA copy number after adjustment for confounding variables including age, body mass index, waist circumference, total cholesterol, HDL-cholesterol, LDL-cholesterol, triglycerides, hs-CRP, serum ferritin, HOMA-IR, K-MMSE, GDS-15, hypertension, diabetes, dyslipidemia, currently smoking, alcohol drinking, and regular exercise.

**Conclusions:**

This study showed that leukocyte mtDNA copy number was positively correlated with leukocyte telomere length in community-dwelling elderly women. Our findings suggest that telomere function may influence mitochondrial function in humans.

## Introduction

Mitochondria are the cellular energy-generation organelles that convert ingested food into adenosine triphosphate (ATP) using the electron transport chain. In this process, reactive oxygen species (ROS) are produced as necessary by-products and can easily lead to mitochondrial DNA damage and respiratory chain impairment [1]. Mitochondria also play an essential role in cell proliferation, differentiation and apoptosis [2]. Functional changes in mitochondria may be associated with aging, age-related conditions or disorders, and age-related loss of vigor [3-5]. Therefore, mitochondrial gene stability and biogenesis are necessary for maintaining the functions of cells, tissues and organs.

Telomeres are repetitive sequences of DNA (TTAGGG) located at the ends of mammalian chromosomes and contribute to the stability and function of chromosomes [6]. Telomere length is influenced not only by birth telomere length as the genetic factor [7] but also by inflammation, oxidative stress, or psychosocial as environmental factors [8-10]. Leukocyte telomere length decreases in length with age, and its shortening is thought to be associated with increased aging-related diseases, such as cardiovascular disease, dementia and type 2 diabetes mellitus, as well as overall mortality [11-14]. Furthermore, telomere length in red blood cells is a strong predictor of lifespan in birds [15]. Therefore, telomere length has been deemed a useful marker for monitoring aging.

Both telomere length and mitochondrial function are generally accepted as being reflective of aging. Recent studies showed that telomere dysfunction including shortening is associated with impaired mitochondrial biogenesis and function, as well as increased ROS level [16]. It has been suggested that the telomere-p53-peroxisome proliferator-activated receptor gamma coactivator (PGC) axis has a direct connection with telomere dysfunction and mitochondrial compromise in a telomere dysfunction mouse model [16].

However, there has been no study about the possible association between telomere and mitochondrial function in humans. Therefore, the purpose of the present study was to determine the relationship between mitochondrial and telomere functions using leukocyte mtDNA copy number and telomere length.

## Patients and Methods

### Ethics Statement

All subjects participated in the study voluntarily, and written informed consent was obtained from each participant. The study complied with the Declaration of Helsinki and the institutional review board of Gangnam Severance Hospital approved this study.

### Study subjects

The Yonsei Aging Study was designed to identify factors related to cognitive function, physical performance and the aging process in community dwelling, otherwise healthy elderly people in Korea [17,18]. In all, 200 elderly people were recruited through public health centers located in the Yangpyung and Ilsan districts in 2008. In this cross-sectional study, we included 129 women over the age of 60 years. The subjects had no previous diagnoses of dementia, Parkinson’s disease, stroke, ischemic heart disease, congestive heart failure, arrhythmia, depression, cancer, thyroid disorders, or chronic renal disease. Patients who were taking estrogen or those with histories of estrogen replacement therapy were excluded. Additionally, individuals with serious cognitive dysfunction (MMSE score ≤10) or functional impairment of Activities of Daily Living and Instrumental Activities of Daily Living (score >0) were also ruled out.

### Assessment of clinical parameters

All subjects completed a lifestyle questionnaire which assessed alcohol consumption, smoking status, physical exercise, current medications, and medication history. Alcohol consumption was defined as the consumption of one or more drinks per week. Patients who reported that they smoked at the time of the study were considered to have a smoking habit. Regular exercise was defined as physical exercise performed for at least 30 min more than three times each week.

One highly trained examiner conducted all of the anthropometric measurements. Body weight was measured to the nearest 0.1 kg using an electronic scale while participants wore light clothing and no shoes. Height was measured to the nearest 0.1 cm using a stadiometer. Waist circumference was measured midway between the lowest rib and the iliac crest while participants stood upright, and hip circumference was measured at the maximal protrusion of the greater trochanter. Body mass index (BMI) was calculated as weight/height^2^ (kg/m^2^). Bioelectrical impedence analysis was used to estimate body fat percentage using an Inbody 3.0 meter (Biospace, Seoul, Korea). Blood pressure was measured in the sitting position after a 10-min rest period.

Biochemical tests were performed on blood samples collected after overnight fasting (>12 hours). Serum levels of fasting glucose, total cholesterol, HDL-cholesterol, triglycerides, and high-sensitivity C-reactive protein (hs-CRP) were measured using an ADVIA 1650 Chemistry System (Siemens, Tarrytown, NY, USA). Low-density lipoprotein (LDL)-cholesterol was calculated using Friedewald’s formula [LDL-cholesterol=total cholesterol − high-density liproprotein (HDL)-cholesterol − (triglycerides/5)] if the serum triglyceride level was below 400 mg/dL. Fasting insulin level was measured by electrochemiluminescence immunoassay (Roche, Indianapolis, IN, USA), and insulin resistance was estimated using the homeostasis model assessment of insulin resistance (HOMA-IR) index [(insulin (µlU/ml) × fasting blood glucose (mg/dl)/18)/22.5]. Ferritin level was assayed using an Immulite 2000 (Siemens, Tarrytown, NY, USA). The hypertension group was defined as patients with systolic BP ≥140 mmHg or diastolic BP ≥90 mmHg or use of anti-hypertensive medication. The diabetes group was defined as participants with fasting blood glucose ≥126 mg/dL or those who used insulin or hypoglycemic medication. The dyslipidemia group was defined as patients with hypercholesterolemia (>240 mg/dL), hypertriglyceridemia (≥150 mg/dL), low HDL-cholesterol (<50 mg/dL), or those using lipid-lowering agents.

### Assessment of depression and cognitive function

For screening of depression, we used the validated Korean version of the short-form Geriatric Depression Scale (GDS-15), which consists of 15 questions [19]. Cognitive function was evaluated with the Korean Mini-Mental State Examination (K-MMSE). Both questionnaires were administered by two experienced family physicians who fully understood their use.

### Measurement of leukocyte mitochondrial DNA (mtDNA) copy number

DNA from peripheral leukocytes was extracted from 1 ml of whole blood using a commercial kit (Qiagen Inc, Valencia, CA, USA). The relative mtDNA copy number was measured using real-time polymerase chain reaction (RT-PCR) with the Light Cycler-Fast Start DNA Master SYBR Green I kit from Roche Molecular Biochemicals (Pleasanton, CA, USA). mtDNA quantity was normalized by simultaneous measurement of the nuclear gene β-globin [20]. Forward and reverse primers for β-globin were 5′-GAAGAGCCAAGGACAGGTAC-3′ and 5′-CAACTTCATCCACGTTCACC-3′, respectively, and forward and reverse primers for the mitochondrial ND1 gene were 5′-AACATACCCATGGCCAACCT-3′ and 5′-AGCGAAGGGTTGTAGTAGCCC-3′, respectively. After denaturation at 95 °C for 300 s, DNA samples were subjected to 40 cycles of incubation at 95 °C for 0.1 s, 58 °C for 6 s, and 72 °C for 18 s. The number of PCR cycles necessary to produce 20 ng of DNA product was defined as the threshold cycle number (Ct), and the mtDNA copy number was calculated using the following equation: relative copy number = 2^ΔCt^ (ΔCt = Ct_β-globin_ − Ct_ND1_).

### Measurement of leukocyte telomere length

Genomic DNA was extracted from whole blood using the G-spin^TM^ Genomic DNA Extraction Kit for Blood (iNtRON Biotechnology Inc., Korea). All DNA samples were diluted to the same concentration (based on ultraviolet (UV) absorbance) and stored at -80°C until use. Leukocyte telomere length was measured as telomere repeat copy number relative to single gene copy number (T/S ratio) by quantitative real-time PCR, as previously described by Cawthon [21]. Real-time PCR was performed using a LightCycler 2.0 (Roche, Mannheim, Germany), and the rate of accumulation of amplified DNA was measured by continuous monitoring with the LightCycler FastStart DNA Master SYBR Green I (Roche Diagnostic, Mannheim, Germany), with MgCl_2_ at a final concentration of 2 mM. The primers for the telomere PCR were 200 nmol/L of 5'-GGTTTTTGAGGGT GAGGGTGAGGGTGAGGGTGAGGGT-3' and 200 nmol/L of 5'-TCCCGACTATCCC TATCCCTATCCCTATCCCTATCC CTA-3'. The primers for the beta-globin PCR were 300 nmol/L of 5’-GCTTCTGACACAACTGTGTTCACTAGC-3' and 500 nmol/L of 5'-CACCAACTTCATCCACGTTCACC-3'. The thermal cycling profile for telomere amplification was 95°C for 10 min followed by 25 cycles of 95°C for 10 s and 58°C for 1 min; the beta-globin amplification was 95°C for 10 min followed by 35 cycles of 95°C for 10 s and 56°C for 15s. Each sample was run in duplicate using 25 ng of DNA per 10 µl reaction. A no-template control was included in each run, and the same calibrator sample was used in all runs to allow comparison of results across runs. A melting curve analysis was performed on every run to verify specificity and identity of the PCR products. Quantitative values were obtained from the Ct value at which a single increase associated with exponential growth of PCR products was detected using LightCycler analysis software. The Ct values were used to calculate the T/S ratio for each sample using the following equation: T/S=2^−ΔCt^ (where ΔCt = Ct_single-copy gene_-Ct_telomere_). The coefficients of variation (CV) of the telomere, single-gene and T/S ratio duplicate assays were <4%, <3%, and <5%, respectively.

### Statistical analyses

Data are presented as mean ± standard deviation (SD) in a normal distribution, median with interquartile range (IQR, 25^th^-75^th^ percentile) in non-normal distribution or number (%) in categorical variables. Insulin, HOMA-IR, hs-CRP, triglycerides, serum ferritin, and mtDNA copy numbers were logarithmically transformed prior to statistical analyses in order to approximate a normal distribution. Pearson correlation coefficients were calculated to evaluate the relationships between mtDNA copy number and the continuous variables. Significance was defined at the 0.05 level of confidence. We performed a stepwise multiple linear regression analysis to exclude the influences of potential confounding variables. Significance for entry into the model used the 0.15 level automatically determined in the stepwise regression. All calculations were performed using the SAS 9.1 statistics package (SAS Institute, Inc., Cary, NC, US).

## Results

The mean age of the participants was 73.74±6.99 years, and the mean log transformed mtDNA copy number and telomere length were 0.63±0.25 and 0.91±0.36, respectively. Table 1 shows the clinical characteristics of the study participants.

**Table 1:** Clinical characteristics of study subjects (N=129).

**Variables**	**Value**
Age (years)	73.74 ± 6.99
Body mass index (kg/m^2^)	25.23 ± 3.25
Waist circumference (cm)	87.87 ± 8.81
**Cardiometabolic parameters**	
Systolic blood pressure (mmHg)	131.04 ± 16.98
Diastolic blood pressure (mmHg)	73.66 ± 10.32
Fasting glucose (mg/dL)	100.38 ± 24.59
Fasting insulin (µIU/mL)	5.91 (4.04-9.21)
HOMA-IR	1.42 (0.90-2.26)
Total cholesterol (mg/dL)	188.62 ± 36.84
High-density lipoprotein cholesterol (mg/dL)	53.43 ± 12.73
Low-density lipoprotein cholesterol (mg/dL)	108.89 ± 35.59
Triglyceride (mg/dL)	110 (89-55)
High-sensitivity C-reactive protein (mg/mL)	0.10 (0.053-0.172)
Ferritin (ng/mL)	76.39 (54.39-110.90)
Log mitochondrial DNA copy number	0.63 ± 0.25	
Telomere length (T/S ratio)	0.91 ± 0.36	
**Mental function**	
Korean mini-mental state examination (score)	24.58 ± 4.18	
Geriatric depression scales-15 (score)	6.31 ± 3.78	
Hypertension	80 (62.02)	
Diabetes	21 (16.28)	
Dyslipidemia	64 (49.61)	
Regular exercise	62 (48.06)	
Alcohol drinking	6 (4.65)	
Current smoking	4 (3.10)	

Note: HOMA-IR; homeostasis model of assessment of insulin resistance.

Regular exercise was defined as physical exercise performed for at least 30 min more than three times each week. Alcohol drinking was defined as the consumption of one or more drinks per week.

Data are expressed as mean ± SD or number (%). Skewed data are expressed as median (25^th^-75^th^ percentile).

**Figure 1:**
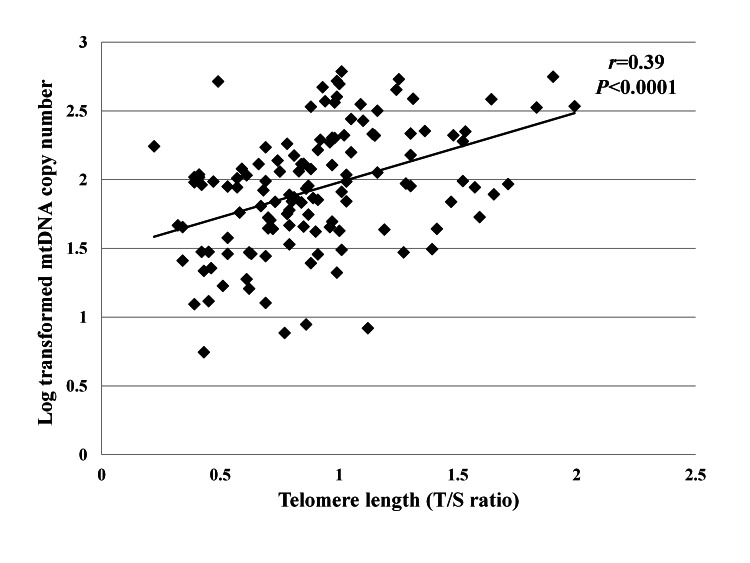
Relatioship between leukocyte mtDNA copy number and telomere length. Values of mtDNA copy number were analyzed after log-transformation. *p*-values were calculated by Pearson’s correlation.

Table 2 shows the associations between leukocyte mtDNA copy number and measured parameters. In univariate analyses, leukocyte mtDNA copy number was positively associated with K-MMSE score (r=0.06, p=0.02). Additionally, leukocyte mtDNA copy number was negatively correlated with GDS-15 score (r=-0.17, p=0.04). Age (r=-0.15, p=0.09), waist circumference (r=-0.16, p=0.07), and serum ferritin level (r=-0.13, p=0.07) tended to be inversely correlated with leukocyte mtDNA copy number, although the relationship was not statistically significant. Figure 1 shows the relationship between leukocyte mtDNA copy number and telomere length (r=0.39, p=<0.0001).

**Table 2:** Correlation between leukocyte mtDNA copy numbers and various parameters.

**Variables**	***r***	***P*-value**
Age	-0.15	0.09
Body mass index	-0.01	0.90
Waist circumference	-0.16	0.07
**Cardiometabolic parameters**		
Systolic blood pressure	-0.07	0.45
Diastolic blood pressure	0.02	0.81
Fasting glucose	-0.05	0.12
Fasting insulin	0.08	0.36
HOMA-IR	-0.12	0.10
Total cholesterol	0.02	0.78
High-density lipoprotein cholesterol	0.13	0.11
Low-density lipoprotein cholesterol	-0.07	0.46
Triglyceride	0.02	0.80
High-sensitivity C-reactive protein	-0.04	0.67
Ferritin	-0.13	0.07
**Mental function**		
Korean mini-mental state examination	0.06	0.02
Geriatric depression scales-15	-0.17	0.04

Note: HOMA-IR; homeostasis model of assessment of insulin resistance.

Coefficients(r) and p-values were calculated by the Pearson correlation model.

Fasting insulin, HOMA-IR, triglyceride, high sensitivity C-reactive protein, and ferritin were analyzed after log-transformation to correct for the skew in the distribution.

Table 3 shows the independent associations between telomere length and mtDNA copy number. The multivariate model explained 16% of the variance of mtDNA copy number by telomere length (β=0.253, p=<0.0001), 3% by current smoking (β=-0.028, p=0.03), 3% by hypertension (β=-0.091, p=0.03), 2% by serum ferritin (β=-0.056, p=0.06), and 1% by waist circumference (β=-0.004, p=0.12) in stepwise multiple regression analysis that included age, BMI, waist circumference, total cholesterol, HDL-cholesterol, LDL-cholesterol, triglycerides, hs-CRP, serum ferritin, HOMA-IR, K-MMSE score, GDS-15 score, hypertension, diabetes, dyslipidemia, current smoking, alcohol drinking, and regular exercise.

**Table 3:** Stepwise multiple regression analysis for leukocyte mtDNA copy number.

		**β coefficient**		**SE**		***F*-value**		***P*-value**	
Telomere length		0.253		0.056		23.12		<0.0001	
Current smoking		-0.028		0.012		5.06		0.03	
Hypertension		-0.091		0.040		4.66		0.03	
Ferritin		-0.056		0.032		3.50		0.06	
Waist circumference		-0.004		0.002		2.47		0.12	

R^2^=0.25. All variables left in the model are significant at the 0.15 level. No other variable met the 0.15 significant level for entry into the model. Variables included in the stepwise model: age, body mass index, waist circumference, total cholesterol, HDL-cholesterol, LDL-cholesterol, triglyceride, hs-CRP, HOMA-IR, K-MMSE, GDS-15, hypertension, diabetes, dyslipidemia, alcohol drinking, regular exercise, current smoking.

Fasting insulin, HOMA-IR, triglyceride, high sensitivity C-reactive protein, and ferritin were analyzed after log-transformation to correct for the skew in the distribution.

## Discussion

This study showed that leukocyte mtDNA copy number was positively correlated with leukocyte telomere length in elderly women. We also found that leukocyte telomere length was an independent factor associated with leukocyte mtDNA copy number after adjusting for potential confounders including age, obesity and inflammatory indices in a stepwise multiple linear regression analysis.

Recently, many studies have shown that a low leukocyte mtDNA copy number is correlated with mitochondrial-related metabolic disorders or conditions, such as insulin resistance [22,23], glucose dysregulation [24], non-alcoholic fatty liver disease [25], elevated homocysteine level [26], and hyperlipidemia [27]. Therefore, leukocyte mtDNA copy number could not only be related with mitochondrial biogenesis reflecting mitochondrial function, but also might be used as a surrogate marker of numerous metabolic diseases associated with mitochondrial dysfunction. Mitochondrial dysfunction may additionally be related to the aging process [1,2,4,5]. The increase of ROS production and the decrease of ATP generation in the mitochondria both play a pivotal role in cellular aging, and mitochondrial dysfunction can lead to aging-associated conditions or disorders such as neurodegenerative diseases, sarcopenia and malignancy [3].

The mitochondrial circular genomes that encode 37 genes are composed only of respiratory complex subunits and some mitochondrial tRNA and rRNA [28]. Therefore, in the regulation of mitochondrial biogenesis as well as various functions including cellular respiration, nuclear proteins play an important role through the expression of nuclear-encoded genes. These proteins which regulate mitochondrial functions include transcription factors such as nuclear respiratory factor (NRF)-1 and peroxisome proliferator-activated receptor (PPAR) α and γ, transcriptional coactivator such as PGC-1α and -1β, and enzymes such as Sirt1 (NAD^+^-dependent deacetylase), AMP-activated protein kinase, and the mammalian target of rapamycin (mTOR, kinase) [28]. Interestingly, a recent study showed that telomeres, the nucleoprotein complexes found at chromosome ends, can influence not only oxidative defense mechanisms, but also mitochondrial function, including biogenesis and metabolism in transcriptomic, molecular, genetic, and functional analyses of various cells or organs such as proliferative and post-mitotic tissues [16]. Further impaired mitochondrial biogenesis and decreased energy production were observed in telomerase-deficient mice with severe telomere dysfunction compared to that in telomerase-deficient mice with largely intact telomeres [16]. It has been suggested that p53 induced by telomere dysfunction and PGCs repressed by the p53 or directly by telomere dysfunction act as potential pathophysiologic mediators between telomere dysfunction and mitochondrial compromise [16]. Therefore, this telomere-p53-PGC-mitochondria axis may explain why shortened telomeres lead to metabolic deterioration related to biological aging.

Caloric restriction (CR), the reduction of total caloric intake while still maintaining adequate nutrition, is the most effective and useful manner to increase the lifespan [29-31] and is also associated with improvement of metabolic compromises and prevention of aging-related disorders [32,33]. The longer lifespan associated with CR is involved in increased mitochondrial mass and respiration [34]. Although it has been reported that proteins, such as Sirt1, play a key role in the interaction of CR and lifespan lengthening [34], the mechanism of longevity extension by CR is not fully understood. A recent study suggested that adult-onset, short-term CR may prevent telomere shortening without increased telomerase activity or reduced oxidative damage in the small intestine and the liver of mice [35]. A conflicting result has also been reported regarding the effects of CR on telomere function. Long-term CR did not influence telomere length in leukocytes or muscles of rhesus monkeys [36]. In humans, further research will be needed to clarify the relationship between CR and telomere function/dynamics. However, the possibility cannot be excluded that improvement of mitochondrial function via preservation of telomere function is involved in other mechanisms of CR-associated health benefits.

Serum ferritin reflects body iron stores and is known to be an index of oxidative stress [37]. It has been reported that increased serum ferritin level is associated with insulin resistance [38], chronic inflammation [39] and metabolic syndrome [40]. Additionally, cigarette smoke contains many oxidants [41], and smokers show an elevated oxidative stress status [42]. In our study, serum ferritin concentration and current cigarette smoking status were inversely correlated with leukocyte mtDNA copy number. These results support the previous findings that oxidative stress can lead to a decrease in mitochondrial biogenesis.

Increasing evidence also indicates that there is a highly significant association between mitochondrial dysfunction and vascular diseases such as hypertension [43-47]. It has been suggested that mitochondrial dysfunction, related to elevated ROS production [43-45], increased mitochondrial Ca^2+^ accumulation [46], and polymorphisms of mitochondria-shaping genes [47], may act as potential pathophysiological mechanisms in hypertension. We also found that patients in the hypertension group consistently showed decreased mtDNA copy numbers in this study.

This study has some limitations. First, it is difficult to identify the mechanisms that underlie the relationship between leukocyte mtDNA copy number and telomere length with a cross-sectional study design. Second, our results may not be generalizable to men or adults because our subjects are only elderly women. Third, the small sample size of our study is another limitation. Although our results showed a positive association between leukocyte mtDNA copy number and telomere length, further study is needed for a valid conclusion. Fourth, we did not examine mitochondrial biogenesis in skeletal muscle, which is generally accepted as the gold standard for evaluation of mitochondrial function. However, repair and regeneration of skeletal muscle, a post-mitotic tissue, may be limited, and most studies relating to the human telomeres use leukocytes, since peripheral blood can be obtained with a relatively noninvasive procedure. It has been suggested that the mtDNA content of the peripheral blood may reflect mtDNA density of muscle and liver tissue in rats [48]. And, data of telomere length and mtDNA copy number, provided in the same resources, could impart coherence in interpretation of results. Finally, we did not measure the levels of oxidative stress and therefore cannot directly investigate the role of oxidative stress as a mediator between leukocyte mtDNA copy number and telomere length. In spite of these limitations, this study was the first to show the relationship between mitochondrial biogenesis and telomere length in humans.

In conclusion, this study showed that leukocyte mtDNA copy number was positively correlated with leukocyte telomere length in community-dwelling elderly women. Our findings suggest that telomere function may influence mitochondrial function in humans. Further studies are needed to clearly examine the associations not only between mitochondrial and telomere function, but also between mitochondrial dysfunction-dependent metabolic disorders and telomere dysfunction.
